# 2D surface optical lattice formed by plasmon polaritons with application to nanometer-scale molecular deposition

**DOI:** 10.1038/s41598-017-08175-8

**Published:** 2017-08-10

**Authors:** Yanning Yin, Supeng Xu, Tao Li, Yaling Yin, Yong Xia, Jianping Yin

**Affiliations:** 1 State Key Laboratory of Precision Spectroscopy, Department of Physics, East China Normal University, Shanghai, 200062 China; 2 National Laboratory of Solid State Microstructures, College of Engineering and Applied Sciences, Nanjing University, Nanjing, 210093 China; 3 NYU-ECNU Institute of Physics at NYU Shanghai, Shanghai, 200062 China

## Abstract

Surface plasmon polaritons, due to their tight spatial confinement and high local intensity, hold great promises in nanofabrication which is beyond the diffraction limit of conventional lithography. Here, we demonstrate theoretically the 2D surface optical lattices based on the surface plasmon polariton interference field, and the potential application to nanometer-scale molecular deposition. We present the different topologies of lattices generated by simple configurations on the substrate. By explicit theoretical derivations, we explain their formation and characteristics including field distribution, periodicity and phase dependence. We conclude that the topologies can not only possess a high stability, but also be dynamically manipulated via changing the polarization of the excitation laser. Nanometer-scale molecular deposition is simulated with these 2D lattices and discussed for improving the deposition resolution. The periodic lattice point with a width resolution of 33.2 nm can be obtained when the fullerene molecular beam is well-collimated. Our study can offer a superior alternative method to fabricate the spatially complicated 2D nanostructures, with the deposition array pitch serving as a reference standard for accurate and traceable metrology of the SI length standard.

## Introduction

Recently, there has been great interest and advancement in nanofabrication with atomic and molecular depositions on solid materials^1–5^. Since the de Broglie wavelength of a thermal atom (or molecule) is small—typically on the order of picometers—due to its relatively low velocity and large mass, the fundamental diffraction effects are not a limiting factor for atomic (or molecular) nanolithography. Also, its sub-optical-wavelength resolution approaches extremely short length scales, bypassing the inherent limitations of conventional lithography. For instance, an one-dimensional (1D) laser standing wave was used to focus an atomic beam deposited on a substrate, first with sodium atoms^6^, and then with chromium^1^, aluminum^7^, ytterbium^8^, and iron atoms^9^ etc. However, this approach is limited to a small selection of atoms amenable to laser cooling, and the laser systems required are much more complex for the two-dimensional (2D) spatially periodic or quasi-periodic patterns, which will require standing wave field constructed and controlled precisely by two or more laser beams. The standing waves created by counter-propagating laser beams can be used to trap ultracold atoms (or molecules) and generate an optical lattice. Atomic (or molecular) ensembles in an optical lattice provide an ideal quantum system where all parameters are controlled, which can be used as model systems for fundamental studies of quantum condensed matter physics^10^, precision measurement^11^, quantum information processing^12^, and so on. The optical lattice created by counter-propagating laser beams, however, should be precisely controlled and carefully preserved, since any tiny fluctuation or unbalance in polarization, phase difference and intensity associated with the laser beams could lead to the instability of the lattice.

On the other hand, a tremendous progress in surface plasmonics has attracted much attention from various fields^13, 14^. Surface plasmon polaritons (SPPs), as a form of surface electromagnetic waves from the collective oscillation of electrons at a metal-dielectric interface^15^, hold promise for a large variety of applications due to their unique characteristics such as the tight spatial confinement and the high local field intensity. Not only have SPPs been a powerful tool in fundamental physics, materials science and nanotechnology^16^, but also have opened up broad prospects for many interdisciplinary fields, with previous studies involving plasmonic detector^17^, plasmonic spin-Hall effect^18^, optical information technology and optoelectronics^13, 14^, surface-enhanced Raman scattering^19, 20^, surface plasmon resonance sensing^21^, subwavelength optics in microscopy and lithography beyond diffraction limit^22^, photonic data storage^23^, shaped SPP beam^24^, controllable excitation of SPPs^25^ and so on. In 2005, Z. Liu *et al*. demonstrated the possibility of the interference of SPP waves used for a new nano-photolithography technique^26^. They also demonstrated experimentally the broad-band 2D manipulation of surface plasmons by shaping the edges of a metallic film and adjusting the parameters of the excitation laser beam^27^. In 2010, Q. Wang *et al*. reported the detection of high-resolution 2D plasmonic spot array formed by subwavelength sized slits illuminated by the incident light with linear polarization using the near-field scanning optical microscope (NSOM)^28^. In 2013, P. Dvořák *et al*. presented experimentally the way to control surface plasmon interference patterns by variation of the slit geometry and by a proper combination of laser beam polarization and inhomogeneous far-field illumination^29^.

Our focus is toward new topologies of lattices with confined and enhanced electric fields, and the dynamical control of the lattice topologies via changing the polarization of the excitation laser. Molecules can be loaded into the lattices and then deposited to form 2D periodic patterns with deposition point or array pitch. As the array pitch fabricated by this technique is directly traceable to the laser wavelength, it has a potential application to serve as a reference standard for accurate and traceable metrology of the SI (International System of Units) length standard. Here, we present a scheme for the realization of highly-stable nanometer-scale optical lattice based on the surface plasmon polariton interference field (SPPIF), which we will refer to as the surface plasmon polariton optical lattice (SPPOL), since the corresponding periodic potential can trap neutral molecules via AC Stark shift^30^. By rigorous and explicit theoretical derivations and computer simulations, we demonstrate the photo-excitation, formation and properties of the 1D and 2D SPPIFs, and describe their feasibility to obtain the different stable patterns of 1D and 2D SPPOLs by means of different structures and excitation light polarizations. We derive the electric filed distributions of 1D and 2D SPPIFs, and investigate the SPPOL’s characteristics including the topology, periodicity and phase dependence. We show that the SPPOLs can possess a high stability in the topologies and intensity distributions since they are excited by only one laser beam with appropriate polarization, in contrast with traditional optical lattice constructed by two or more beams and corresponding reflectors. This scheme also allows the standing wave fields formed by SPPs to be switched easily and modified quickly, offering a favorable flexibility to the nano-deposition technology and allowing more choices of atomic and molecular species in the nanostructures. This technology can be implemented in a direct deposition mode with neutral molecules focused by molecule lenses into an extremely fine spot upon depositing onto a substrate.

## Materials and Methods

To provide an insight into the formation of the 2D surface optical lattice based on the SPPIF and give a rigorous theoretical explanation of the lattice topology and its dynamical manipulation, we first elucidate the basic principle and structure for the photo-excitation of the SPPIF in a simple 1D case and analyze some key parameters related to the features of the SPPOL. Afterwards, the materials and methods used here, including the SPP-generating configuration, calculation and simulation methods, will be extended to the more complicated 2D case.

### Configuration for the formation of SPPIF in 1D structure

As a form of surface electromagnetic waves propagating along a metal-dielectric interface, the electric field of the SPPs can be expressed by

1 $$E={E}_{0}\exp [i({k}_{x}x+{k}_{z}z-\omega t)],$$

where ${k}_{x}$, ${k}_{z}$ are the wave vectors, respectively, and $\omega$ is the frequency of the wave. By solving the Maxwell’s equations for the electromagnetic wave at the interface between semi-infinite metal and dielectric materials with permittivities, ${\varepsilon }_{m}$ and ${\varepsilon }_{d}$ respectively, with the appropriate continuity relation, the dispersion relation for the SPPs can be written as^31^

2 $${k}_{x}=\frac{\omega }{c}{(\frac{{\varepsilon }_{m}{\varepsilon }_{d}}{{\varepsilon }_{m}+{\varepsilon }_{d}})}^{1/2}.$$

**Figure 1:**
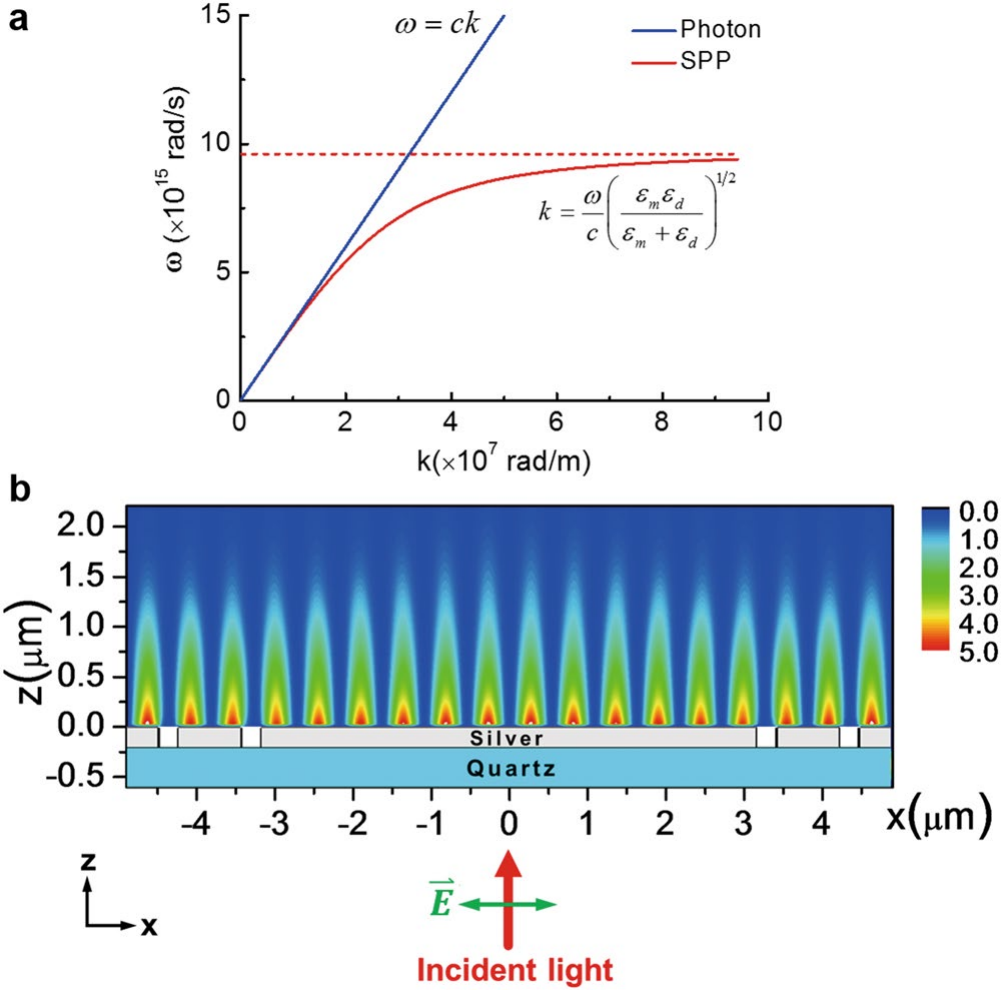
Fundamental photo-excitation of the SPPs and the SPPIF in 1D structure. (**a**) A typical dispersion curve for SPP generated in the interface between silver and air (red curve) and a universally known dispersion line for photon (blue line). As the k increases, at first the SPP behaves like a photon, but then the SPP dispersion relation bends over and reaches an asymptotic limit called the surface plasma frequency given by ${\omega }_{p}/\sqrt{2}$. (**b**) The configuration for the photo-excitation of the SPPIF in 1D case and the resultant interference field. The green double-headed arrow indicates the polarization of the normally incident laser beam (red arrow). The thicknesses of the silver film and the quartz substrate are 200 nm and 400 nm, respectively. The width of the silver slits is 260 nm and the distance between the two slits is 800 nm. The intensity distribution of SPPIF is obtained at an incident excitation light’s electric field of 1 V/m.

According to the above equation, a typical SPP dispersion curve and a universally known dispersion line for photon with $\omega =ck$ are shown in Fig. 1a. Since the wave vector of the SPPs is always larger than that of the light at the same frequency (i.e., smaller wavelength of SPPs), a momentum compensation coupling mechanism must be adopted to photo-excite SPPs effectively. Among several common momentum-matching methods, grating is one convenient and appropriate way since it can provide an additional discrete wave vector related to the grating periodicity to meet the momentum-matching condition. For a 1D grating with a periodicity of d, surface plasmon waves with a wave vector ${k}_{SPP}=2\pi /d$ can be photo-excited by a normally incident light at a proper wavelength according to the conservation of momentum^27, 28^.

The photo-excitation and formation of the SPPIF in 1D case are illustrated in a rather simple configuration shown in Fig. 1b ^32^. The quartz substrate with a thickness of 400 nm is covered with a 200 nm-thick silver film with two double-slits, each of which contains four slits serving as a grating when the periodic boundary condition is adopted. The width of the slits is set to be 260 nm and the distance between the two slits is 800 nm. Once the distance between two double-slits is carefully chosen, a normally incident excitation light at 1095 nm with the polarization along the x direction, that is, perpendicular to the grating slits, can be coupled to generate the SPP waves propagating away from the slits and then form a uniform SPPIF at the middle region of the film.

As shown in Fig. 1b, we numerically calculate the intensity distribution at the cross section of the silver film using the finite difference time domain (FDTD) method. From the parameters and the intensity distribution given in Fig. 1b, it is seen that the incident laser beam from the bottom of the quartz substrate is coupled to generate the SPP waves at 1060 nm, exactly corresponding to the grating constant of 1060 nm. The periodicity of the 1D SPPIF is 530 nm, which is half of the SPP waves.

### Numerical description of the 1D SPPIF

To describe the 1D SPPIF numerically, some key physical parameters must be specified. First, as the permittivity of the metal can be well described by the Drude model, ${\varepsilon }_{m}=1-{\omega }_{p}^{2}/({\omega }^{2}+i\gamma \omega )$, where for silver, the bulk plasmon frequency ${\omega }_{p}=1.37\times {10}^{16}\,\text{rad}/{\rm{s}}$, and the electron collision frequency $\gamma =8.5\times {10}^{13}\text{rad}/{\rm{s}}$ at 1095 nm^33^, the wave vector of the SPPs should be expressed in terms of its real and imaginary components as ${k}_{x}={k^{\prime} }_{x}+i{k^{\prime\prime} }_{x}$, which reflects that the intensity of the SPPs decays while propagating along the surface with a propagation length ${L}_{SPP}={(2{k^{\prime\prime} }_{x})}^{-1}$ (For silver, ${L}_{SPP}\approx 500\,{\rm{\mu }}{\rm{m}}$ at the wavelength $\lambda =1060\,{\rm{nm}}$). Likewise, the electric field falls off evanescently perpendicular to the metal surface with a decay length $\widehat{z}=1/|{k}_{z}|$ (For silver, $\widehat{z}\approx 1.4\,{\rm{\mu }}{\rm{m}}$ at $\lambda =1060\,{\rm{nm}}$). Second, since only the SPPs with the TM mode can exist at the interface^34^, which means that the magnetic field of the SPPs is parallel to the surface and perpendicular to the propagation direction, the electric field of the SPPs has both longitudinal and transverse components, denoted as ${E}_{x}$, ${E}_{z}$ respectively. Comparing the magnitudes of these two components, $|{E}_{z}|/|{E}_{x}|={(-{\varepsilon }_{m}/{\varepsilon }_{d})}^{1/2}\approx 7.9$, it is obvious that ${E}_{z}$ plays a primary role in the formation of the resultant interference pattern. In this sense, the polarization of the SPP waves here can be regarded as along the z direction, that is, perpendicular to the plane of the metal surface. Therefore, the electric field of the 1D SPPs and thus the 1D SPPIF can be described by

3 $${E}_{z}^{SPP}(x,z,t)={E}_{00}{e}^{i({k}_{x}^{^{\prime} }x-\omega t)}{e}^{-x/(2{L}_{SPP})}{e}^{-z/\hat{z}},$$

4 $${E}_{z}^{SPPIF}(x,z,t)={E}_{00}{e}^{-i\omega t}{e}^{-z/\hat{z}}[{e}^{i{k}_{x}^{^{\prime} }x}{e}^{-x/(2{L}_{SPP})}+{e}^{-i{k}_{x}^{^{\prime} }x}{e}^{-(L-x)/(2{L}_{SPP})}],$$

where E_00_ is the amplitude of the electric field of the SPPs, which can be obtained by the numerical result of the simulation based on the FDTD method, and L is the distance between the two double-slits and is set to be an integral multiple of the half-wavelength of the SPPs. As discussed above, the propagation length of the SPPs generated by the configurations here is ${L}_{SPP}\approx 500\,{\rm{\mu }}{\rm{m}}$, which is much larger than L (6360 nm). Under this condition, the Eq. (4) can be approximated as

5 $${E}_{z}^{SPPIF}(x,z,t)=2{E}_{00}{e}^{-i\omega t}{e}^{-z/\hat{z}}\,\cos ({k^{\prime} }_{x}x),$$

which is exactly the expression of an interference standing wave with a periodicity of half of the SPP wavelength.

### Configurations for different types of 2D SPPIFs

**Figure 2:**
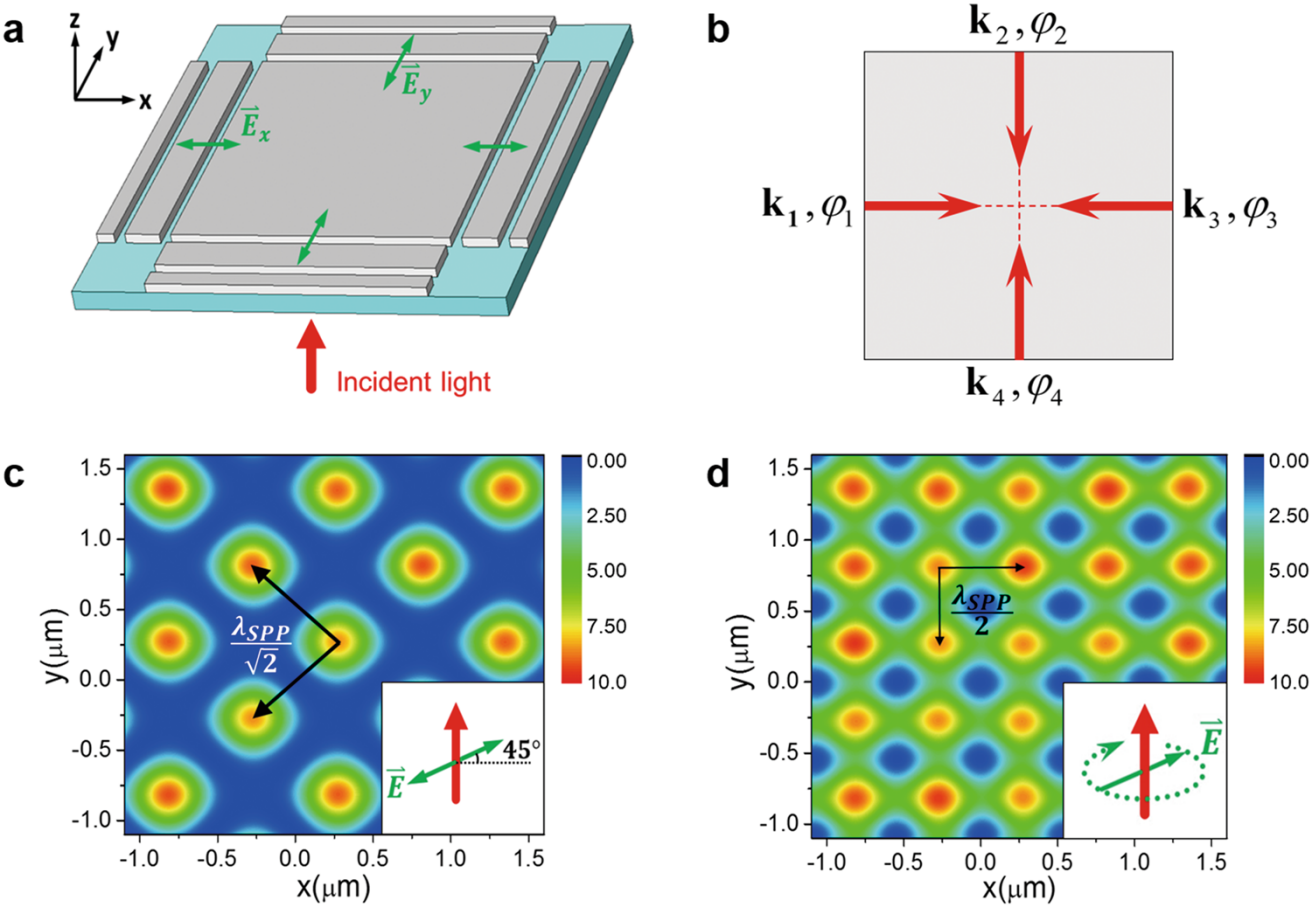
Demonstration of the realization of two different types of 2D SPPIFs. (**a**) The configuration for achieving two different types of 2D SPPIFs. The 2D structure is extended from the 1D case. There are two double-slits in both x and y directions, and the structure is illuminated by a laser beam (red arrow) with linear or circular polarization from the bottom. The thickness of the silver film and the substrate, the size and distance of the slits in x or y direction are the same with those in Fig. 1b. The green double-headed arrows marked with ${\mathop{E}\limits^{\rightharpoonup }}_{x}$ and ${\mathop{E}\limits^{\rightharpoonup }}_{y}$ indicate the vibration directions of the effective electric field components of the incident light that can work in exciting SPPs in that corresponding direction. (**b**) The illustration of the generation of 2D SPPIFs. Four excited SPP waves with wave vectors ${{\bf{k}}}_{{\bf{1}}},{{\bf{k}}}_{2},{{\bf{k}}}_{3},{{\bf{k}}}_{4}$ lying on one plane and phases ${\phi }_{1}={\phi }_{3},$ ${\phi }_{2}={\phi }_{4}={\phi }_{1}+\Delta \phi$ are overlapped with each other, where $\Delta \phi =0$ for incident light of linear polarization and $\Delta \phi =\pi /2$ for circular polarization. The polarization of the SPP waves is linear and out of the plane of intersection. (**c**–**d**) The resultant intensity distribution (at z = 100 nm) of 2D SPPIFs formed at the surface of silver film that can serve as SPPOLs with distinct topologies, the checkerboard lattice for (**c**) with spacing $d={\lambda }_{SPP}/\sqrt{2}$ and the normal square lattice for (**d**) with spacing $d={\lambda }_{SPP}/2$. The inset shows the corresponding incident light with linear polarization for (**c**) and circular polarization for (**d**).

From the discussion on the 1D SPPIF, 2D SPPIF patterns are available by intersecting more than two SPP waves with appropriate polarizations and phase differences. As depicted in Fig. 2, configurations for achieving the different types of 2D SPPIFs and the resultant 2D patterns (intensity distribution on the silver film) are demonstrated. These configurations are readily extended from the structures of the 1D case except that the polarizations of the incident excitation light should be carefully considered to obtain the desired patterns that could serve as different types of optical lattices.

In Fig. 2a, two types of 2D SPPIFs are both created by a configuration consisting of a quartz substrate and a silver film with double-slits in both x and y directions, while the normally incident excitation light can be linearly polarized at an angle of 45 degrees to the x (or y) axis, or circularly polarized. The difference in the excitation light polarization results in the different types of SPPIFs, which will be discussed in the following section.

## Results and Discussion

### Simulation results of different types of 2D SPPIFs

In Fig. 2, the configuration with different excitation light polarization leads to the different types of SPPIFs on the surface of the silver film, which can be regarded as SPPOLs with distinct topologies (here we use “topology” to describe the different intensity patterns of surface optical lattice): the checkerboard lattice in Fig. 2c and the normal square lattice in Fig. 2d, which are quite common in the current optical lattices generated by the overlapping of four laser beams in cold atoms^35^. The explanation for the two different SPPOLs involves the relative phase of the two orthogonal 1D standing waves that lead to the 2D SPPIFs (with respect to each other). Because the SPPs can only be efficiently photo-excited by the incident light with a polarization perpendicular to the slits in the 1D case, the incident light with a diagonally linear polarization or circular one in the 2D cases can both be decomposed into two orthogonal components, one of which is linearly polarized perpendicular to the corresponding slits in the x direction, and the other is linearly polarized perpendicular to the slits in the y direction:

6 $${{\bf{E}}}_{{\bf{l}}{\bf{i}}{\bf{g}}{\bf{h}}{\bf{t}}}={{\bf{E}}}_{{\bf{x}}}+{{\bf{E}}}_{{\bf{y}}}={E}_{x0}\,\cos ({k}_{{\rm{i}}}z-{\omega }_{{\rm{i}}}t){{\bf{e}}}_{{\bf{x}}}+{E}_{y0}\,\cos ({k}_{{\rm{i}}}z-{\omega }_{{\rm{i}}}t+{\rm{\Delta }}\phi ){{\bf{e}}}_{{\bf{y}}},$$

where ${E}_{x0}$, ${E}_{y0}$ are the amplitudes of the components, respectively, and ${k}_{i}$ is the wave vector and ${\omega }_{i}$ is the frequency of the light, and ${\rm{\Delta }}\phi$ is the phase difference of the two components. For instance, the 45-degree linearly polarized incident light is composed of two linearly polarized components with ${E}_{x0}={E}_{y0}$ and $\Delta \phi =0$, while the circularly polarized incident light is composed of two linearly polarized ones with ${E}_{x0}={E}_{y0}$ and $\Delta \phi =\pi /2$. While the component ${{\bf{E}}}_{{\bf{x}}}$ can excite the SPP waves in the x direction and the component ${{\bf{E}}}_{{\bf{y}}}$ can excite the SPP waves in the y direction, the relations of amplitude and phase difference between the two components can be conserved and then transferred to the generated SPP waves in x and y directions.

As illustrated in Fig. 2b, the four excited SPP waves ${{\bf{k}}}_{{\bf{1}}}$, ${{\bf{k}}}_{2}$, ${{\bf{k}}}_{3}=-{{\bf{k}}}_{{\bf{1}}}$ ${{\bf{k}}}_{4}=-{{\bf{k}}}_{2}$ with the same polarization ${{\bf{e}}}_{z}$ and absolute phases ${\phi }_{1},$ ${\phi }_{2}={\phi }_{1}+\Delta \phi ,$ ${\phi }_{3}={\phi }_{1},$ ${\phi }_{4}={\phi }_{2}={\phi }_{1}+\Delta \phi$ intersect with each other and form two orthogonal 1D standing waves. When the incident light is linearly polarized with an angle of 45 degrees to the x (or y) axis, this situation corresponds to the interference of the two 1D standing waves propagating along different axes with a phase difference of $\Delta \phi =0$, where we have $|{{\bf{k}}}_{1}|=|{{\bf{k}}}_{2}|=|{{\bf{k}}}_{3}|=|{{\bf{k}}}_{4}|={{\rm{k}}^{\prime} }_{x}$ and ${\phi }_{1}={\phi }_{2}={\phi }_{3}={\phi }_{4}$. Considering that the polarization of SPP waves is linear and perpendicular to the plane of intersection, the resulting electric field of the 2D SPPIF can be described as

7 $${E}_{z}^{SPPIF}(x,y,z,t)=2{E}_{00}{e}^{-i\omega t}{e}^{-z/\hat{z}}[\cos ({k^{\prime} }_{x}x)+\,\cos ({k^{\prime} }_{x}y)].$$

A so-called checkerboard SPPOL (square lattice oriented along the diagonals) is obtained.

### Change the topology from checkerboard to normal square

It is significant to clarify some characteristics of the SPPIF-based 2D SPPOLs. Periodicity is an important parameter for optical lattice, and here we refer to the spacing between the adjacent maximum points of the intensity as the periodicity, as we will consider the molecules with a larger transition frequency than the SPP frequency (i.e. a red-detuned SPPIF for the molecules) to discuss the potential of the SPPOL. For the SPPIF-based 2D SPPOL with checkerboard topology in Fig. 2c, the spacing is $d={\lambda }_{SPP}/\sqrt{2}\approx 750{\rm{nm}}$ along the diagonals. The topology of the SPPOL can be tuned by shifting the relative phase of the two orthogonal SPP standing waves with respect to each other. By simply changing the polarization of the incident light in an appropriate way, that is, from linear to circular, the lattice can be translated in space and the topology of the lattice can be continuously changed from the checkerboard lattice with spacing ${\lambda }_{SPP}/\sqrt{2}$ to a normal square lattice oriented parallel to the coordinate system with spacing $d={\lambda }_{SPP}/2=530\,{\rm{nm}}$ for $\Delta \phi =\pi /2$, where we have ${\phi }_{1}={\phi }_{3}=0,{\phi }_{2}={\phi }_{4}=\pi /2$, as described in Fig. 2d. Meanwhile, the electric field distribution of this 2D SPPIF can be expressed as

8 $${E}_{z}^{SPPIF}(x,y,z,t)=2{E}_{00}{e}^{-i\omega t}{e}^{-z/\hat{z}}[\cos ({k^{\prime} }_{x}x)+i\,\cos ({k^{\prime} }_{x}y)].$$

Therefore, different types of 2D SPPIFs can be realized by the relative simple configurations, and these SPPIFs can serve as the SPPOLs with different topologies.

It is worth mentioning that, compared to the optical lattices formed by lasers in cold atoms, the SPPOLs have the superiority of extraordinarily stable interference fields because they are actually created by one single laser beam with an appropriate polarization, and the phase difference between two standing waves is inherently steady. The optical lattices by lasers, however, are usually constructed by two or more laser beams controlled by Michelson interferometer to lock the relative phase of these laser beams^35^. Any tiny fluctuation or unbalance in polarization, phase difference or intensity among the laser beams could lead to instability of the lattice. Accordingly, the precise direction and control of the laser beams often necessitate complicated optics, but as for the SPPOLs in our case, these issues can simply be replaced by well-designed slits and a single excitation laser beam.

### Realization of SPPOL with triangular topology

**Figure 3:**
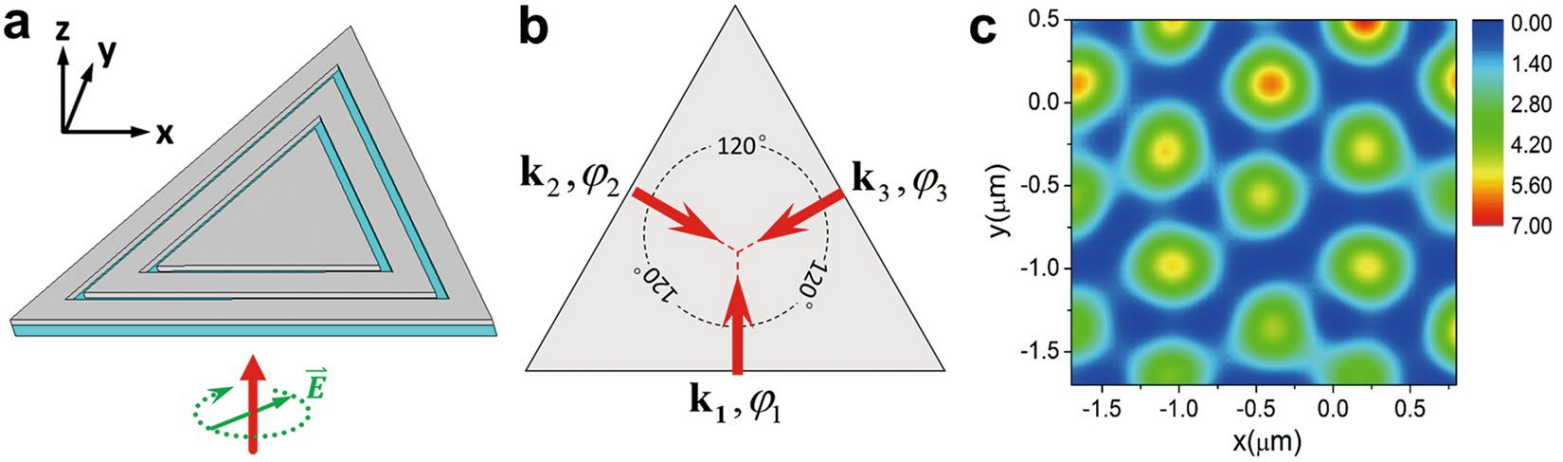
Demonstration of the realization of 2D SPPIF with triangular topology. (**a**) The configuration for achieving the 2D SPPIF that can serve as SPPOL of triangular topology. Three slits are arranged in a shape of equilateral triangle and the incident light is circularly polarized. (**b**) The illustration of the formation of the 2D SPPIF. Three excited SPP waves intersect under an angle of 120° in one plane and their linear polarization points out of the plane. (**c**) The resultant intensity distribution (at z = 100 nm) of the 2D SPPIF which shows a pattern of triangular structure.

Design for realizing SPPOLs with other kinds of topologies is also accessible by carefully changing the arrangements of the slits, such as the triangular topology. A regular 2D triangular lattice is obtained when three SPP waves with equal wavelength are overlapped under 120 degrees in one plane. As shown in Fig. 3a, the structure including three double-slits arranged in a shape of equilateral triangle is irradiated by a laser light with a circular polarization. The three excited SPP waves with wave vector ${{\bf{k}}}_{{\bf{1}}},{{\bf{k}}}_{{\bf{2}}},{{\bf{k}}}_{{\bf{3}}}$ from the different slits can intersect under an angle of 120° above the silver film, as shown in Fig. 3b, which form the SPPIF with an intensity distribution showing a pattern of triangular structure (in Fig. 3c), and that is similar to the optical lattice created by three linearly polarized laser beams^36^.

By defining the wave vectors of the three SPP waves as

9 $${{\bf{k}}}_{{\bf{1}}}={k^{\prime} }_{x}(\begin{array}{c}0\\ 1\end{array}),{{\bf{k}}}_{2}=\frac{{k^{\prime} }_{x}}{2}(\begin{array}{c}\sqrt{3}\\ -1\end{array}),{{\bf{k}}}_{3}=\frac{{k^{\prime} }_{x}}{2}(\begin{array}{c}-\sqrt{3}\\ -1\end{array}),$$

the electric field of the 2D SPPIF with a triangular intensity pattern can be described by

10 $${E}_{z}^{SPPIF}({\bf{r}},z,t)={E}_{00}{e}^{-z/\hat{z}}[\,\cos ({{\bf{k}}}_{{\bf{1}}}\cdot {\bf{r}}-\omega t+{\phi }_{1})+\,\cos ({{\bf{k}}}_{2}\cdot {\bf{r}}-\omega t+{\phi }_{2})+\,\cos ({{\bf{k}}}_{3}\cdot {\bf{r}}-\omega t+{\phi }_{3})],$$

where ${\bf{r}}=(x,y)$ is the position vector in the x-y plane, ${\phi }_{1,2,3}$ are the phases of the three SPP waves. For an incident excitation light with a circular polarization which can be described by Eq. (6) where $\Delta \phi =\pi /2$, the three excited SPP waves can have phase difference calculated to be $\Delta {\phi }_{12}={\phi }_{1}-{\phi }_{2}=\frac{2}{3}\pi$ and $\Delta {\phi }_{23}={\phi }_{2}-{\phi }_{3}=\frac{2}{3}\pi$. Therefore, if we set the phase ${\phi }_{3}=0$, the other two phases would be ${\phi }_{1}=\frac{4}{3}\pi ,{\phi }_{2}=\frac{2}{3}\pi$.

### Application to nanometer-scale 2D molecular deposition

Molecules in a non-resonant optical field will experience a dipole force due to the AC Stark effect. The potential energy and corresponding dipole force are given by^37^

11 $${U}_{dip}({\bf{r}})=-\frac{1}{2{\varepsilon }_{0}c}\mathrm{Re}(\alpha )I({\bf{r}}),$$

12 $${F}_{dip}({\bf{r}})=-\nabla {U}_{dip}=\frac{1}{2{\varepsilon }_{0}c}\mathrm{Re}(\alpha )\nabla I({\bf{r}}),$$

where α is the complex-valued molecule polarizability, and $I({\bf{r}})$ is the field intensity. Molecules in the red-detuned SPPIF are brought with an attractive interaction potential, so the molecules will be attracted to and thus accumulate on the antinodes of the SPPIFs.

Nanofabrication is the one of the best applications of optical lattices to date. One promising application of our 2D SPPIFs is the nanometer-scale atomic or molecular deposition, which offers a simple and superior method to fabricate spatially complicated 2D nanostructures for the potential nanotechnology applications. As discussed above, molecules in the SPPIFs can be manipulated by the dipole force that points to the antinodes, which actually enables each period of the SPPIFs to act as a molecular lens to converge the molecules spatially and eventually deposit the molecules periodically. Based on the intensity distribution of our different 2D SPPIFs, molecular deposition with different patterns can be achieved.

The dynamic process of the molecular nano-deposition using the various 2D SPPIFs is performed by the Monte Carlo trajectory simulation method^38^. The fullerene C_60_ is selected as a prototype molecule amenable to nanometer-scale deposition by the non-dissipative dipole force in the SPPIFs since the C_60_ moleucle has been widely studied and utilized in molecular optics and nanomaterials science^39, 40^. In the Monte Carlo simulation, molecules are generated with randomized velocities in a Gaussian distribution which matches the measured forward and transverse velocity distributions of a molecule beam source^41^. A cold C_60_ molecular beam with a most probable longitudinal velocity of approximately 65 m/s, a velocity spread of ${\rm{\Delta }}v=20\,{\rm{m}}/{\rm{s}}$ and a transverse divergence angle of 1 mrad is normally incident on the silver film illuminated by a commercial parametric oscillation (OPO) laser beam with an intensity of $5.0\times {10}^{8}{\text{W}/\text{cm}}^{2}$ from the bottom to generate the desired 2D SPPIFs^32^. The three different structures in Figs 2 and 3 have been employed to obtain the different deposition patterns. The size and parameters of the structures used in our simulation are the same as the corresponding structures in Figs 2 and 3 except for the much greater incident laser intensity.

### Deposition patterns and the improvement of deposition resolution

**Figure 4:**
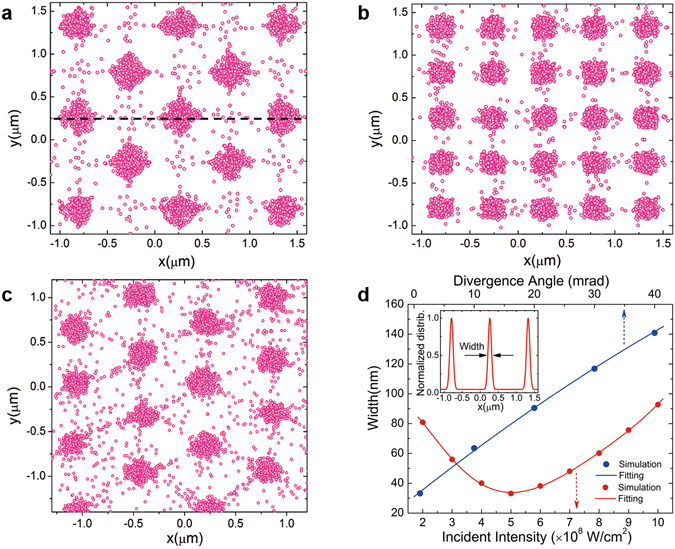
Molecular deposition patterns and the dependence of the width of the deposition point. (**a**–**c**) Deposited molecule distributions of different patterns by the Monte Carlo simulation method, using the three different structures shown in Figs 2c,d and 3c. (**d**) The typical dependence of the width of the deposition point on the light intensity of the incident laser beam (red dots) and on the divergence angle of the molecular beam (blue dots). The red or blue curve is the fitting of the simulation data. The inset shows the normalized 1D density distribution of the deposited molecules along the dash line shown in Fig. 4a and the width is defined as the FWHM of the distribution.

The ultimate deposited molecule distributions of different patterns are shown in Fig. 4a–c, which apparently show that the majority of the molecules are deposited at the antinodes of the 2D SPPIFs, comparing to the intensity distribution of SPPIFs shown in Figs 2c–d and 3c. However, a small fraction of molecules fail to accumulate at the antinodes as a consequence of their large initial transverse velocity along with a high longitudinal speed, indicating that the brighter and cleaner deposition point arrays of different patterns can be achieved by using a colder and better-collimated molecular beam, which can be obtained by the magnetic or electric hexapole field or geometrical skimmer method in the experiment. Then we study the dependence of the width of the deposition point, which is defined in the inset of Fig. 4d, on the incident light intensity, where we take the deposited distribution in Fig. 4a as a typical example.

The simulated results of width versus different incident intensities are shown in red dots and fitting curve in Fig. 4d, which show that the deposition width falls to ~33.2 nm when the incident light intensity is $5.0\times {10}^{8}{\text{W}/\text{cm}}^{2}$. At this optimized incident light intensity, the relation between the deposition width and the transverse divergence angle of the molecular beam is also shown in blue dots and fitting curve in Fig. 4d, which verifies the necessity of a well-collimated molecular beam to yield the fine deposition point arrays. However, when the structures of the silver film or the parameters of the molecular beam are invariable in the realistic experiment, the optimal incident light intensity can be chosen to acquire the narrowest deposition width and thus improve the deposition resolution. The process can be much localized with the minimal scattering, and this technology, due to the excellent long-range spatial coherence, can be achieved with great dimensional accuracy over a large area of the substrate while combining laser focusing of molecules in an interference pattern.

Note that here we have selected the molecule that has no metallic features for the deposition in the SPPIFs. But the pattern of the SPPIFs would be modified if metal atoms are deposited on the surface of the silver film in our configuration, since the deposition process actually coats the silver film periodically with the metal elements, which are thus not recommended for deposition with the SPPIFs.

In addition, the pitch (or spacing) of the highly regular array of deposited points can be traced directly to the wavelength of the standing wave. The pitch fabricated by this technique has a good periodicity, uniformity, spatially coherent consistency and stability with clear fringes and high resolution. The incident laser beam can be a stable frequency source, and the modern stabilization methods make it easy to achieve the highly accurate wavelength or frequency. The connection between laser-focused molecular deposition and incident laser frequency thus opens the possibility of creating a nanoscale length standard up to a highly accurate, constant, physically measurable quantity. The standing wave has a periodicity that is essentially as well determined as the stabilized laser frequency, and this degree of certainty transfers nearly perfectly to the deposited structure, resulting in an inherently well-characterized pitch^42, 43^. Furthermore, our scheme allows the standing wave fields formed by SPPs to be switched easily and modified quickly, offering a favorable flexibility to the deposition points of different patterns.

## Conclusion

In conclusion, we have demonstrated the photo-excitation, formation and properties of the 1D and 2D SPPIFs in intensity, polarization and phase dependence, as well as their feasibility to obtain different stable patterns of 2D SPPOLs by means of different structures and excitation light polarizations. The SPPOL’s characteristics including the topology, periodicity and phase dependence are also investigated. The SPPOLs can possess a high stability in both the topology and the intensity distribution because they are actually photo-excited by a single laser beam with an appropriate polarization. One promising application of our 2D SPPIFs, that is, nanometer-scale molecular deposition, is simulated and discussed for improving the deposition resolution. The periodic lattice point with a width resolution of 33.2 nm can be obtained when the fullerene molecular beam is well-collimated. As the SPPIFs can be switched easily and modified quickly, our scheme offers a favorable flexibility to nano-deposition technology and should allow more molecular and atomic species to be used for the nanostructures, which can be fabricated in a clean, resist-free environment without damage to the underlying substrate. The molecules can be loaded into the lattices and then deposited to form 2D periodic patterns with deposition point or array pitch to serve as a reference standard for accurate and traceable metrology of the SI length standard.
